# Activation of Macrophages by Oligomeric Proteins of Different Size and Origin

**DOI:** 10.1155/2018/7501985

**Published:** 2018-11-18

**Authors:** Indrė Dalgėdienė, Asta Lučiūnaitė, Aurelija Žvirblienė

**Affiliations:** Institute of Biotechnology, Life Sciences Center, Vilnius University, Vilnius LT-10257, Lithuania

## Abstract

Activation of macrophages is one of the key processes in generating the immune response against pathogens or misfolded/aggregated otherwise unharmful host's proteins. Antigens and their immune complexes (IC) may shape macrophage phenotype in various directions. Data on the impact of protein structure during inflammation are evident; however, some separate steps of this process involving changes in macrophage phenotype are not fully understood. Our aim was to investigate the phenotype of macrophages after activation with different oligomeric proteins and their IC. We have used amyloid beta (A*β*_1–42_) that plays a role in neurodegenerative inflammation as a model of host-associated protein and three oligomeric viral antigens as pathogen-associated proteins. Murine cell lines J774, BV-2, and macrophage primary cell culture were treated with oligomeric proteins and their IC. After 48 h, expression of surface markers F4/80, CD68, CD86, and CD206 and secreted cytokines IL-10, IL-12, IL-23, and TNF-*α* was analysed. A*β*_1–42_ oligomers stimulated expression of both inflammatory and anti-inflammatory molecules; however, fibrils induced less intense expression of markers investigated as compared to small and large oligomers. Two out of three viral oligomeric proteins induced the inflammatory response of macrophages. Data suggest that macrophage activation pattern depends on the origin, size, and structure of oligomeric proteins.

## 1. Introduction

Macrophages play a key role in defending organism from various pathogens; however, they also can participate in chronic inflammatory process as seen in certain pathologies such as Alzheimer's disease (AD), multiple sclerosis, or sarcoidosis [1, 2]. It is convenient to differentiate these cells after activation into inflammatory, alternatively activated or otherwise polarised macrophages using M1/M2 phenotype system [3]. Macrophages have been described as classically activated or M1 and alternatively activated or M2 based on their gene expression signatures, surface molecules, and secretion of immune mediators. Lipopolysaccharides (LPS) and cytokines produced by Th1 lymphocytes, such as IFN-*γ*, induce phenotype M1. These macrophages secrete inflammatory cytokines such as IL-6, IL-12, and TNF-*α*. Th2 lymphocyte cytokines, such as IL-4 or/and IL-13, induce phenotype M2 and macrophages secrete anti-inflammatory molecules such as IL-10 and TGF-*β* [4]. However, depending on the microenvironment, macrophages may express various activation molecules that do not fit into the current classification [5]. In addition, classical macrophage populations may be undetectable after cell activation with nonclassical activation patterns.

Studies investigating how macrophages respond to the shape and size of the activation agents report that properties of activators can determine not only the efficiency of phagocytosis [6] but also influence the activation threshold and cell phenotype [7, 8]. Consequently, macrophages of certain phenotypes can be responsible for the course of the diseases or lead to improvement and recovery from the illness [9, 10]. In these examples, the activators were polymeric nanoparticles [6], graphene oxide [8], or protamine-RNA particles [7]. However, none of this tackles the questions how would macrophages respond to the size and shape of protein oligomers which are naturally abundant in the organism or present during infections. For example, measles virus circulating in the bloodstream primarily targets monocytes and lymphocytes during viremia, before infecting a variety of target organs. However, under certain infection complications, measles virus can evade the immune response and enter CNS causing various neuropathologies [11]. Viremia of measles virus manifests in large amounts of virus oligomeric N protein erupting from infected cells, its spreading in the organism and activating immune cells [12, 13]. Therefore, viral oligomeric proteins might be one of many potential macrophage polarization factors.

Another example of oligomeric proteins are amyloid beta (A*β*) aggregates that are involved in the pathogenesis of AD. It is ascertained that A*β* oligomers and fibrils stimulate microglia cells in a different manner [14, 15]. However, varying results leave an open question which macrophage profiles are induced by various oligomeric structures of A*β*. In addition, there were numerous attempts to eliminate A*β* aggregates or at least dissociate them using antibodies via passive or active immunization not only in animal model systems [16] but also in third phase clinical trials as well [17]. Unfortunately, the acute side effects of this therapy such as meningoencephalomyelitis revealed that the immune response to these interventions is not fully understood [18]. After immunotherapy, one of the possible A*β* clearance mechanisms is through Fc receptor-mediated phagocytosis. It was shown in animal models that immune complexes (IC) triggered microglial cells to clear A*β* burden through Fc receptor-mediated phagocytosis and subsequently A*β* aggregates were degraded by the same cells [16]. Macrophages/microglia not only remove pathogenic microorganisms, their toxins, and noxious agents opsonized with antibodies but also send activation signals to other cells of the immune system [19]. Therefore, these phagocytes might fail to neutralize A*β* and their IC and instead propagate even more severe inflammation in their surroundings. Studies were carried out to elucidate the processes of microglia activation with IC composed of A*β*. Induction of inflammatory response in microglia cells was described [20]. Despite the assumed neutralizing effects of anti-A*β* antibodies, the increased neurotoxicity of IC was observed [21]. Nevertheless, a positive effect of anti-A*β* antibodies in microglia activation was demonstrated [22]. These controversial data suggest that not all factors playing a role in macrophage activation with IC are identified.

In the present paper, we investigated the influence of host-associated proteins, A*β*_1–42_ oligomers of various structures and pathogen-derived proteins, viral oligomeric proteins, on macrophage phenotype. We compared the effects of different oligomeric proteins in the *in vitro* model systems of murine macrophages. In both groups of naturally abundant and pathogen-derived protein models, we demonstrated that small, large oligomers and fibrils induce different macrophage activation profiles. In certain cases, the nonclassical phenotype of macrophages was observed. The current study demonstrates the importance of antigen size and structure in macrophage activation.

## 2. Materials and Methods

### 2.1. Cell Lines

Cell line J774 was kindly provided by prof. Vilmantė Borutaitė (Lithuanian University of Health Sciences, Kaunas, Lithuania). Cells were propagated in DMEM + 10% FBS + 2 mM L-glutamine + 100 *μ*g/ml gentamicin and were split twice a week by ratio 1 : 5 to 1 : 10 using scraper.

Cell line BV-2 was purchased from the Interlab Cell Line Collection (ICLC, Italy), accession number: ICLC ATL03001. Cells were propagated in DMEM + 10% FBS + 2 mM L-glutamine + 100 *μ*g/ml gentamicin and were split every 2-3 days by ratio 1 : 5 to 1 : 10 using scraper.

### 2.2. Macrophage Primary Cell Culture

Mouse macrophage primary cell cultures were prepared as described previously [23]. Procedures with BALB/c mice were performed at Vilnius University Life Science Center animal facility (Vilnius, Lithuania) in accordance with EU legislation (State Food and Veterinary Service permission No. LT 61-13-004). Spleens were separated from the abdominal cavity of 6- to 8-week-old female BALB/c mice, crushed, and filtered through a 100 *μ*m nylon strainer. Lysis buffer was used to remove red blood cells and cell suspension was prepared. Then, splenic cells were analysed by flow cytometry to determine the count of CD11b^+^ cells. CD11b^+^ cells were cultured at a density of 0.3 × 10^6^ per well for 5 days in RPMI 1640 + 10% FBS + 2 mM L-glutamine + 100 *μ*g/ml gentamicin and 10 ng/ml M-CSF (Invitrogen, USA) in 12-well plates, at 37°C with 5% of CO_2_. For macrophage activation on day 5^th^, cells were stimulated with LPS (50 ng/ml) and IFN-*γ* (20 ng/ml) or IL-4 (20 ng/ml) and IL-13 (20 ng/ml) to obtain either M1 or M2 phenotype, respectively. After 48 h of incubation, cell culture supernatants were collected and stored at −20°C for further cytokine analysis. The cells were harvested by trypsin 0.05% and 0.02% EDTA (Biochrom, Germany) for flow cytometry analysis.

### 2.3. Preparation of Amyloid Beta Aggregates

Various A*β*_1–42_ forms (oligomers and fibrils) were generated as described previously [24]. Briefly, small and large oligomers were prepared by dissolving 1 mg of A*β*_1–42_ peptide (Covance, USA) in 400 *μ*l hexafluoroisopropanol (HFIP) for 30 min at room temperature (RT) and 5 min sonication was used. Twenty-five *μ*l of the resulting seedless A*β*_1–42_ peptide solution was added to 225 *μ*l H_2_O in a siliconized Eppendorf tube (small oligomers) or nonsiliconized Eppendorf tube (large oligomers). After 20 min of incubation at RT, the samples were centrifuged for 15 min at 12,000 rpm, the supernatant was transferred to a new siliconized or nonsiliconized tube, and the HFIP was evaporated. To obtain small 1–4 nm-sized oligomers, the sample was incubated in a closed tube for 24 h at 20°C with slight agitation. To obtain large 4–10 nm sized oligomers, the sample was incubated in a closed tube for 24 h at 20°C with slight stirring using polytetrafluoroethylene-coated magnet. Fibrils were formed by the protocol in which the aqueous peptide solution obtained after evaporation of HFIP was incubated for 7 days at RT [24]. The size and morphology of the preparations of A*β*_1–42_ oligomers and fibrils were characterized by atomic force microscopy (AFM) as described [24]. A*β*_1–42_ peptide used as a negative control was solubilized in HFIP just before treatment of the cells and added to cell growth medium.

### 2.4. Viral Proteins

Macrophages were activated with recombinant viral proteins representing various oligomeric shapes and forms. WU polyomavirus recombinant major capsid protein VP1 (WUPyV rVP1, 39.9 kDa) forms spherical oligomers—virus-like particles (VLPs)—containing up to 360 monomers as described previously [25]. Measles virus recombinant nucleocapsid protein (MeV rN, 58.0 kDa) forms fibrillar filamentous structures as described previously [26, 27]. Human metapneumovirus recombinant nucleocapsid protein (HMPV rN, 43.5 kDa) forms small structures, mostly circular oligomers as described previously [28]. All virus proteins were expressed in yeast expression system and purified by CsCl density gradient centrifugation [25, 26, 28].

### 2.5. Monoclonal Antibodies and Immune Complexes

For macrophage activation with IC, antigen-specific monoclonal antibodies purified via protein A column were used. Murine monoclonal antibody against N-terminal epitope of A*β*_1–42_ (clone #11E12, subtype IgG2b) was generated by hybridoma technology after immunization of BALB/c mice with A*β*_1–42_ oligomers of 1-2 nm size [29]. Murine monoclonal antibodies against recombinant oligomeric proteins WUPyV rVP1 (clone #11D2, IgG1; clone #12F8, IgG2a; clone #5H10, IgG2b), HMPV rN (clone #11D10, IgG1; clone #11H5, IgG2a; clone #4A2, IgG2b), and MeV rN (clone #22G2, IgG1; clone #7C11, IgG2a; clone #10F7, IgG2b) were described previously [25, 27, 28].

Prior to treatment of cells with IC, the respective antigens and antibodies were mixed in cell culture medium and incubated for 30 min at 37°C. Molar concentrations of the antigens and the respective antibodies used throughout all the experiments are indicated in Table 1 unless stated otherwise. All IC consisting of A*β*_1–42_ oligomers and #11E12 were used in molar ratio 10 : 1 [21], and all IC formed from recombinant virus protein oligomers and the respective monoclonal antibodies were used in molar ratio 1 : 1 unless stated otherwise. In the case of A*β*_1–42_, the molar ratio 10 : 1 was used to increase the accessibility of antibodies to A*β*_1–42_ oligomers because A*β*_1–42_ peptide is small (4.5 kDa) in comparison to mouse antibody molecule (160 kDa).

### 2.6. Flow Cytometry

To evaluate the expression of cell surface and intracellular markers, flow cytometric analysis was performed. Briefly, macrophages after activation with various factors were collected, washed, and resuspended in staining buffer (1% FBS, 0.1% NaN_3_ in PBS). In the case of macrophage primary cell cultures, the cells were detached using 0.05% trypsin and 0.02% EDTA solution. The cells were incubated with trypsin/EDTA solution for 10 min and then trypsin was inhibited with 10% FBS. After washing with staining buffer, the cell surface receptors Fc*γ*RIII (CD16) and FcγRII (CD32) were blocked with TruStain fcX™ (anti-mouse CD16/32) Antibody solution (BioLegend, USA). The cells were incubated with fluorophore labelled antibodies to the respective cell-surface markers for 30 min at 4°C. After washing twice with staining buffer, cells were fixed and permeabilized using Fixation/Permeabilization solution (BD Cytofix/Cytoperm Fixation Permeabilization Kit, BD Biosciences, USA) for 20 min at 4°C. Cells were stained for intracellular markers for 30 min at 4°C. After washing twice with Permeabilization solution (BD Cytofix/Cytoperm Fixation Permeabilization Kit, BD Biosciences, USA), the cells were resuspended in staining solution. Antibodies used in this study include rat anti-mouse CD11b-FITC (clone M1/70, Life Technologies, USA), F4/80-APC (clone BM8, Life Technologies, USA), CD68-PE (clone FA-11, BioLegend, USA), CD86-PE (clone GL1, Life Technologies, USA), and CD206-PE/Cy7 (clone C068C2, BioLegend, USA). The antibody conjugates were used with paired isotype controls. Flow cytometry data was acquired on a CyFlow Space flow cytometer (Sysmex Partec, Germany) and analysed using FlowJo software (FlowJo, LLC, USA). The normalized median fluorescence intensity (NMFI) was calculated by dividing marker median fluorescence intensity (MFI) of treated cells by the MFI of untreated cells for every independent experiment.

### 2.7. Quantitation of Cytokines in Cell Culture Supernatants

ELISA kits for the measurement of cytokine—IL-10, IL-12p40/IL-23p40, IL-12p70, and TNF-*α*—levels in cell culture supernatants were used (ELISA Ready-SET-Go!, eBioscience, USA). ELISA kits are based on sandwich immunoassay technique. Supernatants were used either undiluted or diluted up to 1 : 200. All procedures were performed according to manufacturers' protocols. In the last step, 3,3′,5,5′-tetramethylbenzidine (TMB) substrate solution was added to each well. The plates were monitored for 15 min for colour development, the reaction in wells was stopped with H_2_SO_4_ 3.6% solution, and the wells were read at 450 nm using Multiskan GO microplate spectrophotometer (Thermo Fisher Scientific Oy, Finland). A standard curve was generated from cytokine standard, and the cytokine concentration in the samples was calculated. Concentration of IL-23 in culture supernatants was calculated by subtracting IL-12p70 concentration from IL-12p40/IL-23p40 concentration.

### 2.8. Statistical Analysis

All statistical analyses were performed with GraphPad Prism 6.0 (GraphPad Software Inc., La Jolla, CA). The data are represented as the mean ± SD. Data was analysed using unpaired Student's *t*-test. Differences with *p* value less than 0.05 were considered to be statistically significant.

## 3. Results

### 3.1. Macrophage Model Systems

To determine the profile of macrophage activation, we used M1/M2 phenotype system and markers consistent with it. The selected model systems for macrophages—cell lines J774, BV-2, and monocyte-derived primary macrophage culture—responded accordingly after treatment with classical M1-inducing factors IFN-*γ*/LPS and classical M2-inducing factors IL-4/IL-13 (Table 2).

### 3.2. Various A*β*_1–42_ Structures and Their IC Induced M1-Like Polarization of J774 Cell Line

**Figure 1:**
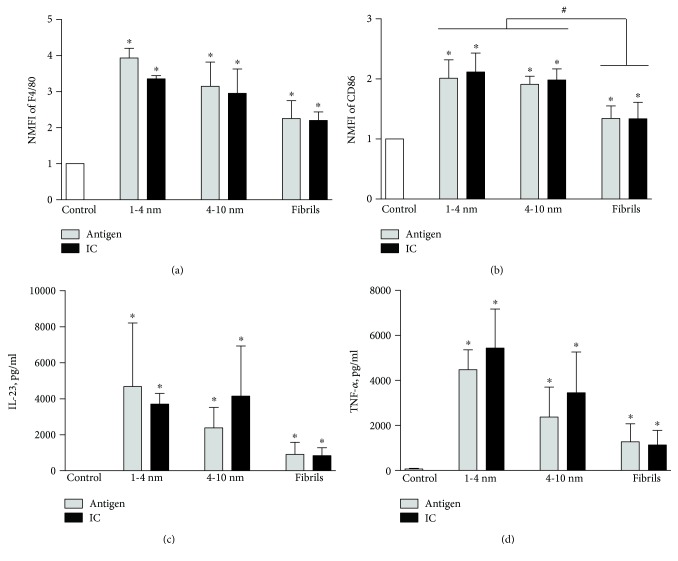
Changes in the expression of cellular markers and cytokine secretion by J774 cell line activated with oligomeric A*β*_1–42_ and their IC. Cells and cell culture supernatants were collected after 48 h of treatment with either small (1–4 nm), large (4–10 nm), or fibrillar A*β*_1–42_ oligomers or their IC and investigated either by flow cytometry (cell-bound markers F4/80 and CD86) or ELISA (cytokines IL-23 and TNF-*α*). As a negative control, untreated J774 cells were examined. (a) and (b) represent normalized median fluorescence intensity (NMFI) of M1 phenotype markers F4/80 and CD86. (c) and (d) represent concentrations of M1-related cytokines IL-23 and TNF-*α*. ^∗^*p* < 0.05 compared with control and #*p* < 0.05 compared between the groups using Student's *t*-test. The bars represent mean ± SD, *n* = 3 − 4 independent experiments.

To establish an appropriate model system for analysing the influence of different A*β*_1–42_ structures and their IC on the activation of phagocytic cells, the response of cell line J774 to the oligomeric proteins was investigated. Treatment of J774 cells for 48 h with A*β*_1–42_ oligomers, fibrils, or their IC induced changes in the expression of several surface-bound and secreted cellular markers. A*β*_1–42_ peptide alone used as a negative control had no effect on the expression of these markers (data not shown). In all cases, we have observed an increase in the expression of surface markers F4/80 and CD86 and an enhanced secretion of cytokines IL-23 and TNF-*α* (Figure 1). Moreover, the increase in the expression of CD86, IL-23, and TNF-*α* but not F4/80 was higher after treatment with small and large A*β*_1–42_ oligomers and their IC as compared to A*β*_1–42_ fibrils and their IC. Meanwhile, we did not observe any changes in the expression of markers CD68 and CD206 or secretion of cytokines IL-12 and IL-10. This indicates that J774 cell line after treatment with various A*β*_1–42_ structures and their IC displays cellular markers consistent with M1 phenotype. Moreover, small and large A*β*_1–42_ oligomers and their IC tend to induce more evident M1 phenotype than A*β*_1–42_ fibrils or their IC.

### 3.3. Various A*β*_1–42_ Structures and Their IC Induced Both M1 and M2 Properties in Primary Macrophage Culture

**Figure 2:**
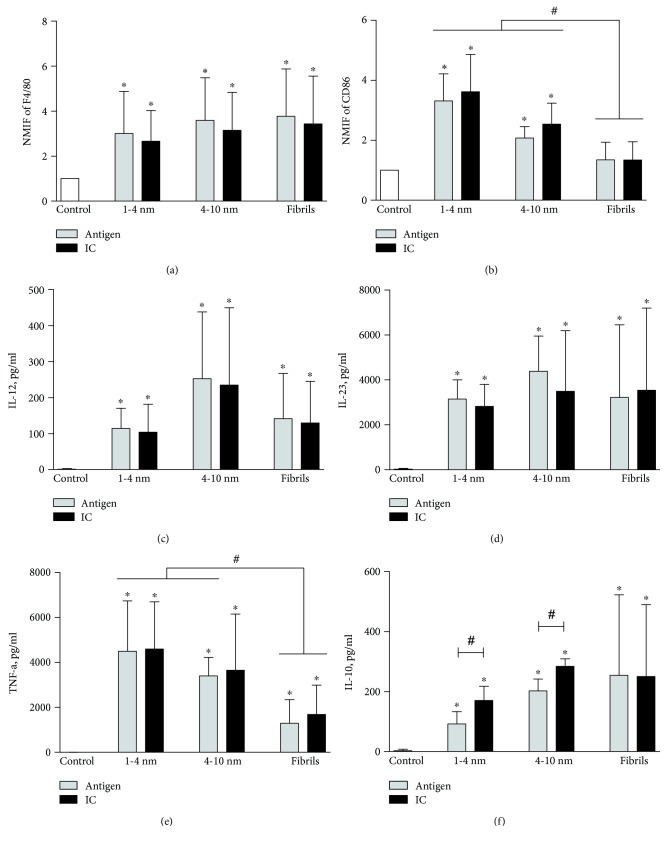
Changes in the expression of cellular markers and cytokine secretion by primary macrophage culture activated with oligomeric A*β*_1–42_ and their IC. Cells and cell culture supernatants were collected after 48 h of treatment with either small (1–4 nm), large (4–10 nm), or fibrillar A*β*_1–42_ oligomers or their IC and investigated either by flow cytometry (cell-bound markers F4/80 and CD86) or ELISA (cytokines IL-12, IL-23, TNF-*α*, and IL-10). As a negative control, untreated cells were examined. (a) and (b) represent normalized median fluorescence intensity (NMFI) of M1 phenotype markers F4/80 and CD86. (c), (d), and (e) represent concentrations of M1-related cytokines IL-12, IL-23, and TNF-*α*. (f) represents concentrations of M2-related cytokine IL-10. ^∗^*p* < 0.05 compared with control and #*p* < 0.05compared inside the group or between groups using Student's *t*-test. The bars represent mean ± SD, *n* = 3 − 4 independent experiments.

The second model system used in our study was primary culture of mouse monocyte-derived macrophages. After activation of primary macrophage culture with A*β*_1–42_ oligomers, fibrils, or their IC, changes in the expression of both M1 and M2 markers were detected. A*β*_1–42_ peptide alone had no effect on the expression of cellular markers (data not shown). In all cases, we have observed an increase in the expression of F4/80 (Figure 2(a)) as well as cytokines IL-12, IL-23, and TNF-*α* (Figures 2(c)–2(e)). Moreover, small and large A*β*_1–42_ oligomers and their IC induced higher secretion levels of TNF-*α* as compared to fibrils. These A*β*_1–42_ oligomers also increased expression of CD86. In contrast, fibrils and their IC had no effect on the expression of this cellular marker (Figure 2(b)). Altogether, these results confirm that A*β*_1–42_ oligomers and their IC induce M1-like phenotype of primary macrophages. Moreover, after macrophage treatment with A*β*_1–42_ oligomers and their IC, we have observed an increase in M2-related marker IL-10 (Figure 2(f)). Secretion level of this cytokine was even higher when macrophages were treated with IC of small and large oligomers as compared to the respective small and large oligomeric proteins alone. These results indicate that A*β*_1–42_ structures and their IC induced the expression of markers consistent with both M1-like and M2-like phenotypes of macrophages.

### 3.4. Various A*β*_1–42_ Structures and Their IC Induced Inferior Activation in BV-2 Cells Compared to Monocyte-Derived Macrophages

After examining how phenotypes of monocyte-derived macrophages depend on A*β*_1–42_ size, form, and their IC, we asked if the same phenotypes would present after activating microglia cells in the *in vitro* model system with various A*β*_1–42._ BV-2 cell line was chosen as a widely used model to investigate various aspects of neuroinflammatory phenomena [30]. After treatment of BV-2 cells with various A*β*_1–42_ structures and their IC under the same conditions as J774 and macrophage primary cell culture, elevated secretion of the M1 marker TNF-*α* was detected in case of large oligomers, their IC, fibrils, and also their IC (data not shown). In case of small A*β*_1–42_ oligomers and their IC, we also observed variations in TNF-*α* secretion but the statistical significance was not reached. Changes of other markers were not observed despite the ability of this cell line to respond to the classical activators and cytokines (Table 1). This indicates that BV-2 phenotype is shifted slightly towards the M1 phenotype after treatment with certain A*β*_1–42_ structures and their IC.

As BV-2 cell line was less responsive to the nonclassical activators as compared to J774 cells and primary macrophage culture, we have excluded BV-2 cell line from further activation experiments involving viral oligomeric proteins.

### 3.5. Different Viral Oligomeric Proteins and Their IC Induce Different Phenotypes of Macrophage Model Systems Compared to A*β*_1–42_ Structures

Results with A*β*_1–42_ represent only a part of a bigger picture on how different antigens or their structural characteristics along with IC may shape the phenotype of macrophages. In addition, A*β*_1–42_ is a protein naturally present in the organism. In order to investigate how mouse macrophages would respond to pathogen-derived oligomeric proteins, we exploited models of various viral proteins. These proteins form different oligomeric structures which serve as examples of foreign antigens of different shape and size. Recombinant VP1 protein of WU polyomavirus (WUPyV rVP1) forms a spherical structure of 40 nm in diameter—virus-like particle (VLP) [25]. Recombinant nucleocapsid protein of human metapneumovirus (HMPV rN) forms circular structures and is of 25 nm in diameter [28], and recombinant nucleocapsid protein of measles virus (MeV rN) also forms circular structures which further form long filamentous structures of 15–20 nm in diameter and various length [27]. In addition, we had a possibility to use various subclasses of antibodies while preparing IC and activating the macrophages with them in order to investigate if the subclasses of IgG have any influence on the manifestation of certain phenotypes.

**Figure 3:**
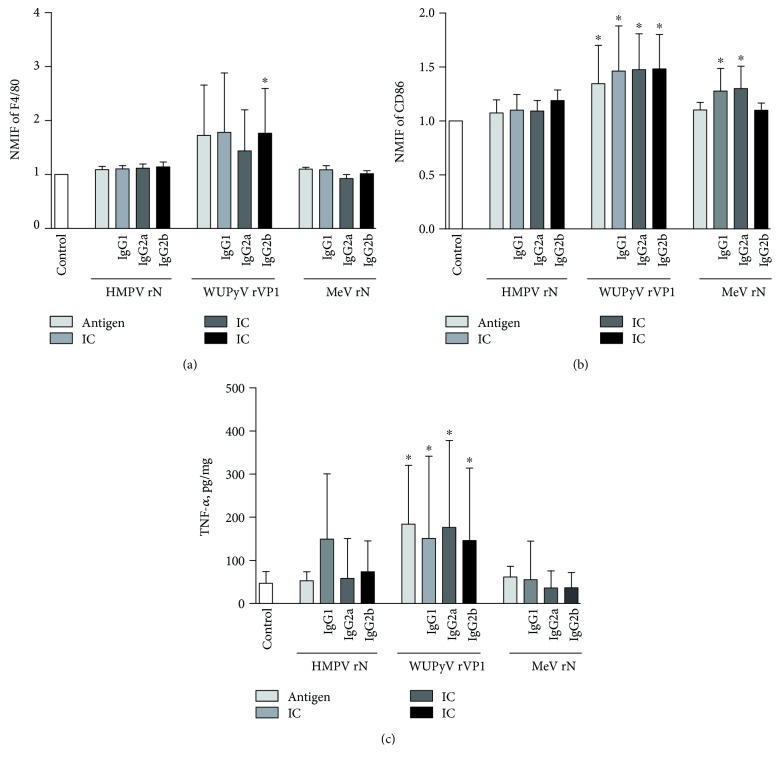
Changes in the expression of cellular markers and cytokine secretion by J774 cell line activated with viral oligomeric proteins and their IC. Cells and cell culture supernatants were collected after 48 h of treatment with either HMPV rN, WUPyV rVP1, MeV rN, or their IC and investigated either by flow cytometry (cell-bound markers F4/80 and CD86) or ELISA (cytokine TNF-*α*). As a negative control, untreated cells were examined. (a) and (b) represent normalized median fluorescence intensity (NMFI) of M1 phenotype markers F4/80 and CD86. (c) represents concentration of M1-related cytokine TNF-*α*. ^∗^*p* < 0.05 compared with control. The bars represent mean ± SD, *n* = 3 − 4 independent experiments.

After treatment of J774 cells for 48 h with annular HMPV rN and their IC, we did not observe any changes in the expression of M1/M2 markers. The effects of spherical oligomeric proteins WUPyV rVP1 and their IC on J774 cells manifested in the increase of F4/80 expression in the case of WUPyV rVP1:IgG2b only (Figure 3(a)). An increase in the expression of M1 marker CD86 as well as TNF-*α* was observed in all cases of WUPyV rVP1 (Figures 3(b) and 3(c)). WUPyV rVP1 did not have any effects on the expression of any other investigated cellular markers. An increase in the expression of M1 marker CD86 appeared after treatment of J774 cells with MeV rN IC containing IgG1 and IgG2a subclasses of IgG antibodies. MeV rN and their IC did not affect the expression of any other markers. Hence, MeV rN IC and WUPyV rVP1 along with their IC had mild effects on J774 macrophage polarization which may be of M1 manner, while HMPV rN and their IC had no significant impact on the phenotype of J774 cells. The subclass of the antibody had a slight impact only in cases of MeV rN:IgG1 and MeV rN:IgG2a.

**Figure 4:**
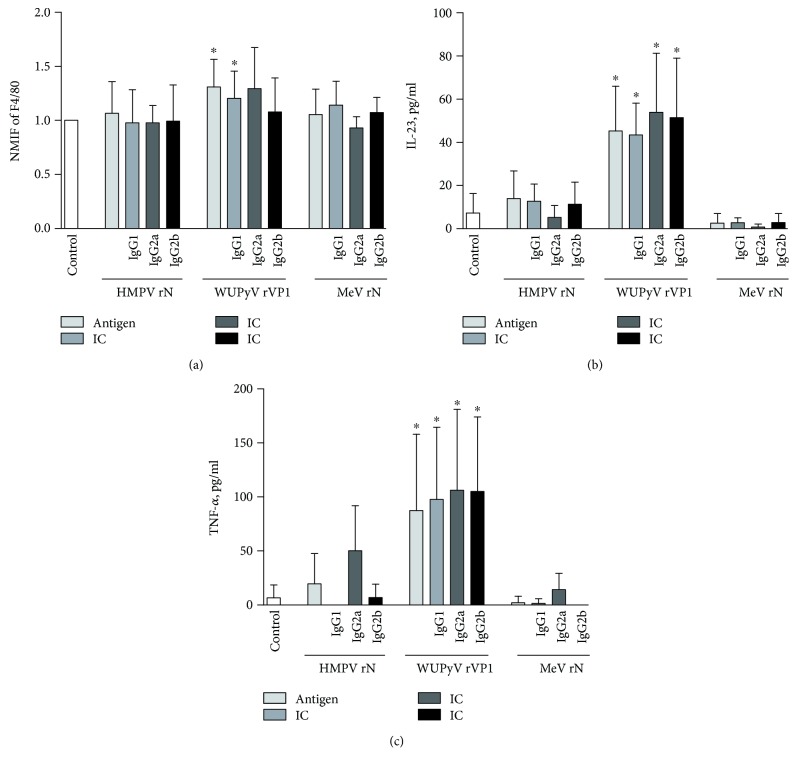
Changes in the expression of cellular markers and cytokine secretion by primary macrophage culture activated with viral oligomeric proteins and their IC. Cells and cell culture supernatants were collected after 48 h of treatment with either HMPV rN, WUPyV rVP1, MeV rN, or their IC and investigated either by flow cytometry (cell-bound markers F4/80 and CD86) or ELISA (cytokine TNF-*α*). As a negative control, untreated cells were examined. (a) and (b) represent normalized median fluorescence intensity (NMFI) of M1 phenotype markers F4/80 and CD86. (c) represents concentration of M1-related cytokine TNF-*α*. ^∗^*p* < 0.05 compared with control. The bars represent mean ± SD, *n* = 3 − 4 independent experiments.

The model system of primary monocyte-derived macrophages also responded to activation only with certain viral oligomeric protein structures and their IC. In the case of circular HMPV rN protein, no effects on activation or the expression of M1/M2 markers were observed (Figure 4). Spherical WUPyV rVP1 and WUPyV rVP1:IgG1 induced an increase in the expression of F4/80 marker and secretion of M1 markers IL-23 and TNF-*α*. IC formed of WUPyV rVP1 and IgG2a or IgG2b caused higher secretion of M1 marker IL-23 only (Figures 4(b) and 4(c)). Fibrillar structures of MeV rN and their IC did not affect any changes of the investigated markers. Overall, these results demonstrate that activation effects of primary monocyte-derived macrophages in case of viral proteins are present only in case of spherical oligomers of WUPyV rVP1 and their IC. All of them shift the macrophages towards the M1 phenotype. Other proteins and their IC had no significant effects reflecting macrophage polarization.

Summarizing the data on viral antigens, large spherical oligomers of viral proteins induced the most significant response in both macrophage model systems. After the activation with 40 nm-sized virus-like particles, J774 cell line as well as macrophage primary cell culture gained properties consistent with M1 phenotype. In case of virus-derived fibrils, only cell line J774 responded to the activation, and changes in cellular markers were observed exclusively with IC. Meanwhile, small annular structures had no effects in both macrophage model systems. Thus, macrophage polarization was dependent on the size and shape of oligomeric viral proteins.

## 4. Discussion

There are many factors determining how macrophages effect their extracellular environment and cells they are in contact with. One of important factors is macrophage phenotype in terms of classical (M1) or alternative polarization (M2) and variations of them [31]. Certain phenotypes may eliminate the activity of pathogenic molecules and their aggregates through phagocytosis but also may play an important role in immunoregulation, while other phenotypes may lead to local and systemic inflammation and tissue damage.

In the current study, we investigated what phenotypes of macrophages are induced after activating them with various A*β*_1–42_ structures and whether these antigens in complex with antibodies would cause the same or different effects. In addition, we examined if these tendencies apply to the phenotype of macrophages activated with pathogen-derived proteins of different origin, namely, viral oligomeric proteins. Three *in vitro* macrophage model systems were used in this study: macrophage cell line J774, microglia cell line BV-2, and primary cell culture of spleen monocyte-derived macrophages.

After J774 cell line activation with various A*β*_1–42_ structures, the rise in the expression of cellular marker F4/80 indicated an efficient cell activation with A*β*_1–42_ oligomers and their IC. F4/80 is assigned to EGF-TM7 (epidermal growth factor seven-transmembrane spanning cell surface molecules) family which function is related to cell adhesion and migration [32]. F4/80 biological function is unknown but the variation in its expression level is related to the physiological statement of the cell [33]. We have detected changes of F4/80 expression after macrophage treatment with classical activation factors IFN-*γ*/LPS (M1) or IL-4/IL-13 (M2). Therefore, we assume that the observed changes in the expression level of this molecule show overall cell activation. As our results indicate, treatment with various oligomeric proteins also increases the expression of F4/80 in macrophages. Cell activation was confirmed with other investigated markers. We have detected an increased expression of M1-related markers CD86, IL-23, and TNF-*α* after J774 treatment with A*β*_1–42_ and their IC. Small and large oligomers and their IC induced higher expression of these markers as compared to fibrils. On the contrary, there was no change in the expression of M2 markers CD206 and IL-10. These data indicate that A*β*_1–42_ oligomers and their IC induce the inflammatory phenotype M1 of macrophages. Our results also show the impact of different oligomeric structure of A*β*_1–42_ on macrophage activation. A similar tendency was observed with primary monocyte-derived macrophages. After the activation of spleen monocyte-derived macrophages with various A*β*_1–42_ structures, an increase in the expression of both M1 and M2 markers was detected. The rise in the expression of F4/80 shows cell activation with A*β*_1–42_ oligomers and their IC. Changes of CD86, IL-12, IL-23, and TNF-*α* expression show the shift towards the M1-like phenotype. The expression levels of CD86 and TNF-*α* were lower after macrophage treatment with fibrils as compared to small and large oligomers of A*β*_1–42_. This emphasizes the importance of A*β*_1–42_ structure in macrophage activation. In contrast to J774 cell line, we also observed an increase in IL-10 secretion after the treatment of primary macrophages with all oligomeric forms of A*β*_1–42_ and A*β*_1–42_ IC. This indicates the generation of macrophages with M2 properties. Furthermore, A*β*_1–42_ IC consisting of small and large oligomers induced higher secretion of IL-10 than oligomeric proteins alone. Higher levels of IL-10 may indicate a negative impact of IC on the phagocytic degradation of A*β* oligomers [34]. In addition, these findings may indicate a positive correlation between macrophage activation levels depending on A*β*_1–42_ size. Previous studies on rat mixed neuronal-glial cultures indicate that the neurotoxicity of A*β*_1–42_ structures depends on their size and shape [21, 24]. Also, our results are in line with other studies where A*β* were shown to induce secretion of proinflammatory cytokines IL-12/IL-23 and TNF-*α* in macrophages/microglia [35, 36]. In addition, higher levels of these cytokines were detected in blood or cerebrospinal fluid samples of AD patients [37, 38].

Previous studies were carried out to elucidate microglia activation with IC composed of A*β*. In one study, microglia activation and secretion of proinflammatory cytokines TNF-*α* and IL-6 were detected after cell treatment with IC [22]. In addition, microglia released more inflammatory cytokines after cell treatment with IC compared to A*β*. In line with these findings, we also detected higher levels of proinflammatory cytokines TNF-*α* and IL-12/IL-23 after activation of monocyte-derived macrophages with A*β*_1–42_ and their IC. However, we did not observe differences in the levels of proinflammatory cytokines when comparing A*β*_1–42_ alone and IC. In contrast, we have detected higher secretion of IL-10 after cell activation with IC consisting of small and large A*β*_1–42_ oligomers. This disagreement between previous studies and the current study might indicate differences between cell model systems used.

Since we have observed a strong response of peripheral macrophages to A*β*_1–42_ oligomers and their IC, we also have investigated their impact on microglia activation. For this purpose, microglia BV-2 cell line was used. We identified the expression of certain cell activation markers after BV-2 activation with LPS/IFN-*γ* and IL-4/IL-13. Changes in cell activation marker F4/80, M1 markers CD86 and TNF-*α*, and M2 marker CD206 were also detected. However, the pattern of BV-2 activation with A*β*_1–42_ oligomers and their IC was different from that observed with J774 cell line and primary monocyte-derived macrophages. After treatment of BV-2 under the same conditions with A*β*_1–42_ and their IC, only TNF-*α* secretion has been changed. In addition, the secretion of this cytokine was considerably lower as compared to monocyte-derived macrophages. These observations indicate that microglia is less responsive to nonclassical activation signals as compared to macrophage cell line or monocyte-derived macrophages. There are more studies showing that A*β* did not induce the release of proinflammatory mediators in microglia commonly induced by lipopolysaccharides [15]. This indicates that A*β* oligomers induce milder microglia activation pattern compared to classical activation patterns. It was also shown that microglia poorly respond to A*β*_1–42_ oligomers or A*β*_1–40_ fibrils [39, 40].

Our study demonstrates the generation of nonclassical macrophage phenotype after cell treatment with A*β*_1–42_. There are many studies showing different activation states of macrophages in response to various antigens. Moreover, in certain cases, especially during disease, an atypical activation pattern of macrophages can appear. These cells can display both M1- and M2-associated gene transcription patterns that do not match the prevailing M1/M2 model [41, 42]. In addition, tissues may contain mixed macrophage subsets with a wide spectrum of activation states [43]. Indeed, our data from different model systems of macrophages treated with different oligomeric proteins indicate that A*β*_1–42_ microenvironment can induce expression of both M1- and M2-related molecules. As for viral oligomeric proteins, macrophages demonstrated only mild M1 activation patterns compared to A*β*_1–42._ Moreover, viral oligomeric proteins did not induce expression of M2-related markers. This can be explained by the well-documented ability of A*β* oligomers to bind to certain cell surface receptors such as TLR and trigger signalling events leading to cell activation [44]. In contrast, data on the presence of specific cellular receptors for viral proteins—MeV N, HMPV N, and WUPyV VP1—on macrophages are rather limited. We have observed a clear correlation between the structure and size of viral proteins and macrophage activation pattern. We investigated the effects of viral proteins representing fibrillar, spherical, or annular oligomeric structures and demonstrated that their size and structure are important factors of macrophage activation. Only spherical oligomeric proteins of WUPyV rVP1 representing 40 nm-sized VLPs induced macrophage activation and their polarization towards M1-like phenotype. In contrast to A*β*_1–42_ oligomers, viral oligomeric proteins induced considerably lower activation signals in macrophages as indicated by lower expression levels of M1-related molecules.

Besides cell-bound activation markers, we have investigated secretion levels of proinflammatory cytokines TNF-*α* and IL-23 in response to macrophage treatment with oligomeric proteins. An increased release of TNF-*α* after treatment of macrophages with small, large, and fibrillar A*β*_1–42_ and their IC was detected. We have also demonstrated that A*β*_1–42_ structural properties are important for this cytokine secretion: A*β*_1–42_ fibrils induced lower secretion of TNF-*α* compared to small and large A*β*_1–42_ oligomers. There are studies showing that neither A*β* oligomers nor fibrils caused TNF-*α* release in primary microglia cell culture [45]. In line with these findings, we have detected low levels of TNF-*α* release by BV-2 cells after their treatment with A*β*_1–42_. Viral oligomeric antigens used in our study were weak inducers of TNF-*α* expression by macrophages. Recombinant N proteins of MeN and HMPV did not induce changes in TNF-*α* secretion in macrophages. It is known that MeV infection downregulates TNF-*α* secretion in monocytes [46]. HMPV infection also reduces TNF-*α* secretion in nasal washes compared to respiratory syncytial virus (RSV) and influenza virus [47]. Data on macrophage-mediated TNF-*α* secretion after their activation with VP1 proteins that form VLPs is limited. Previous study investigating how recombinant VP1 VLPs of human enterovirus 71 induce cytokine response in spleen lymphocyte cultures of an immunised mouse indicate that VLPs caused up to 80 ng/ml TNF-*α* secretion [48]. However, this does not enable to compare VLP effects observed in our study since the cell types are different. Therefore, further investigation would be necessary to determine how recombinant viral proteins mimic the effects of native viral capsids in macrophage activation.

IL-12 is one of the commonly known inflammatory cytokines. Despite its wide coverage in literature as a key player in immune-mediated inflammatory responses [49], we have observed an enhanced IL-12 secretion only in one cell model system when primary macrophage culture secreted this cytokine after activation with all types of A*β*_1–42_ oligomers. Moreover, the highest IL-12 secretion level was observed during cell activation with large A*β*_1–42_ oligomers. Previous studies investigating how microglia respond to different forms of A*β*_1–42_ also indicate that A*β* plaque induces IL-12 secretion [50].

Other inflammatory marker showing macrophage activation with A*β*_1–42_ oligomers and their IC is cytokine IL-23. It is known that IL-23 stimulates differentiation of Th17 cells involved in inflammatory responses [51]. Aberrant regulation of IL-23 expression is related to various inflammatory diseases such as AD. Th17 can also enter the CNS and cause neuronal death through Fas signalling pathway and tissue damage [52]. In our study, all three forms of A*β*_1–42_ along with their IC induced the secretion of IL-23 in J774 and primary macrophage culture. A*β*_1–42_ fibrils and IC induced lower IL-23 levels in J774 cells. Our data indicate that IL-23 secreted after macrophage activation with small and large A*β*_1–42_ might be responsible for the neuronal death and tissue damage observed in previous studies [53].

An opposite effect has cytokine IL-10. It is known that IL-10 has a negative impact on the degradation of phagocytosed A*β* particles [34]. Elevated IL-10 amounts reduce A*β* clearance by mononuclear phagocytes and microglia. According to recent studies with mouse models, the main reason why A*β* aggregates are not degraded by phagocytes is the activity of anti-inflammatory cytokines such as IL-10 [54]. In our research, we have detected higher IL-10 release after macrophage treatment with IC composed of large and small A*β*_1–42_ oligomers compared to oligomers alone. Usually, during AD the proinflammatory molecules are secreted together with anti-inflammatory cytokines [55]. Our results also show an increase in IL-10 secretion after macrophage treatment with A*β*_1–42_ along with inflammatory cytokines such as TNF-*α* or IL-23. In addition, higher IL-10 levels detected after cell treatment with IC compared to A*β*_1–42_ alone bring up a question whether certain immunotherapy strategies involving antibodies may be an efficient treatment mode. During viral infections, some viruses also exploit the benefits of IL-10 for hijacking the immune response [56]. However, the existing experimental data does not implicate viral oligomeric proteins responsible for that. We also did not observe any changes in IL-10 secretion in our model systems after treating them with recombinant viral N proteins of measles and human metapneumovirus or recombinant VP1 protein of WU polyomavirus.

While investigating the influence of antibody subtype in the IC on the macrophage phenotype, we detected cell activation only with certain IC. Results with J774 cells show that IC composed of fibrillar MeV rN oligomers induced cell activation. In addition, J774 activation was observed only with IgG1 and IgG2a subtypes. We also detected cell activation with IC composed of WUPyV rVP1 VLPs, but the activation signal was similar to VLPs alone. Therefore, it is unknown whether oligomers and their IC give the same activations signal or this signal is induced by oligomeric proteins alone. When a multivalent antigen is bound to at least two antibody molecules, the signal induced by the IC occurs by cross-linking of the FcR [57, 58]. This explains why only in certain cases we have detected cell activation with IC. For example, HMPV rN forms small annular oligomers while WUPyV rVP1 and MeV rN consist of many monomers, which means that they expose considerably more epitopes and there is a high probability that more antibodies will bind to the same oligomer. It was demonstrated in previous studies that IC size starting from 0.5 *μ*m up to 2 *μ*m induced the most evident phagocytosis [59]. However, the previously investigated IC size range does not overlap with our protein oligomer models which are in 15–40 nm scale. Consequently, structural properties of activation agents are important and should be taken into account while planning immunotherapy or other research related to the modulation of the immune response.

Summarizing, we have explored a variety of host-associated or pathogen-derived oligomeric proteins as well as three different *in vitro* macrophage model systems and demonstrated that macrophage activation pattern differs depending on the origin and structure of oligomeric proteins leading to either inflammatory or anti-inflammatory phenotype of macrophages.

## 5. Conclusions

The activation of macrophages with oligomeric proteins and their IC leads to different phenotypes depending on the origin, size, and shape of oligomeric proteins and/or the type of cells activated. Our study indicates that A*β*_1–42_ oligomers of various structures—fibrils, large and small oligomers—induce simultaneous expression of inflammatory and anti-inflammatory molecules in macrophages while viral oligomeric proteins induce predominantly an inflammatory response. Also, A*β*_1–42_ oligomeric structures induced more intense cell activation pattern as compared to viral oligomeric proteins. Interestingly, higher IL-10 expression levels after cell treatment with small and large A*β*_1–42_ IC was observed as compared to oligomers alone. This may indicate the importance of A*β*_1–42_ structures on the immune regulation. We have demonstrated that deviations in M1 and M2 phenotypes are possible when macrophages are activated with nonclassical activators such as oligomeric proteins. These findings might be important for further development of vaccination and immunotherapy strategies.

## Figures and Tables

**Table 1:** Concentrations of antigens and the respective antibodies used throughout all the experiments.

Antigen	c [*μ*mol/l]	
		Antibody
A*β*_1–42_ small oligomers	1.0	0.1
A*β*_1–42_ large oligomers	1.0	0.1
A*β*_1–42_ fibrils	1.0	0.1
HMPV rN	0.1	0.1
WUPyV rVP1	0.1	0.1
MeV rN	0.1	0.1

**Table 2:** Expression changes of cell-bound and secreted markers in macrophage cell model systems after treatment with M1- or M2-inducing factors.

Marker	J774		Macrophage primary cell culture		BV-2	
	M1	M2	M1	M2	M1	M2
F4/80	High	High	High	High	High	High
CD68	High	—	—	—	High	—
CD86	High	*Low*	High	*Low*	High	—
IL-12	—	—	High	—	—	—
IL-23	High	—	High	—	—	—
TNF-*α*	High	—	High	—	—	*Low*
IL-10	—	—	—	—	—	—
CD206	—	High	—	High	—	High

M1: cells treated with IFN-*γ*/LPS; M2: cells treated with IL-4/IL-13; high: expression higher than in untreated cells; *low*: expression lower than in untreated cells; —: no changes in expression levels compared to untreated cells.

## Data Availability

The data used to support the findings of this study are included within the article.

## Abbreviations

A*β*: Amyloid beta
AD: Alzheimer's disease
IC: Immune complex (complexes)
HMPV: Human metapnaumovirus
MeV: Measles virus
MFI: Median fluorescence intensity
NMFI: Normalized median fluorescence intensity
rN: Recombinant nucleocapsid protein
rVP1: Recombinant major capsid protein
VLP: Virus-like particle
WUPyV: WU polyoma virus.
